# Physiological and Differential Proteomic Analyses of Imitation Drought Stress Response in *Sorghum bicolor* Root at the Seedling Stage

**DOI:** 10.3390/ijms21239174

**Published:** 2020-12-01

**Authors:** Hongbing Li, Yulin Li, Qingbo Ke, Sang-Soo Kwak, Suiqi Zhang, Xiping Deng

**Affiliations:** 1 State Key Laboratory of Soil Erosion and Dryland Farming on the Loess Plateau, Northwest A & F University, Xianyang 712100, China; qbke@nwafu.edu.cn; 2 State Key Laboratory of Soil Erosion and Dryland Farming on the Loess Plateau, Institute of Soil and Water Conservation, Chinese Academy of Science and Ministry of Water Resources Section, Xianyang 712100, China; sqzhang@ms.iswc.ac.cn (S.Z.); dengxp@ms.iswc.ac.cn (X.D.); 3 College of Life Science, Northwest A & F University, Xianyang 712100, China; liyulin@nwafu.edu.cn; 4 Plant Systems Engineering Research Center, Korea Research Institute of Bioscience and Biotechnology (KRIBB), Daejeon 34141, Korea; sskwak@kribb.re.kr

**Keywords:** antioxidant enzyme, osmotic substance, proteome analysis, MALDI-TOF-TOF-MS, drought tolerance, molecular basis

## Abstract

Drought is one of the most important constraints on the growth and productivity of many crops, including sorghum. However, as a primary sensing organ, the plant root response to drought has not been well documented at the proteomic level. In the present study, we compared physiological alteration and differential accumulation of proteins in the roots of sorghum (*Sorghum bicolor*) inbred line BT×623 response to Polyethylene Glycol (PEG)-induced drought stress at the seedling stage. Drought stress (up to 24 h after PEG treatment) resulted in increased accumulation of reactive oxygen species (ROS) and subsequent lipid peroxidation. The proline content was increased in drought-stressed plants. The physiological mechanism of sorghum root response to drought was attributed to the elimination of harmful free radicals and to the alleviation of oxidative stress via the synergistic action of antioxidant enzymes, such as superoxide dismutase, peroxidase, and polyphenol oxidase. The high-resolution proteome map demonstrated significant variations in about 65 protein spots detected on Coomassie Brilliant Blue-stained 2-DE gels. Of these, 52 protein spots were identified by matrix-assisted laser desorption/ionization time-of-flight tandem mass spectrometry (MALDI-TOF-TOF MS) representing 49 unique proteins; the levels of 43 protein spots were increased, and 22 were decreased under drought condition. The proteins identified in this study are involved in a variety of cellular functions, including carbohydrate and energy metabolism, antioxidant and defense response, protein synthesis/processing/degradation, transcriptional regulation, amino acid biosynthesis, and nitrogen metabolism, which contribute jointly to the molecular mechanism of outstanding drought tolerance in sorghum plants. Analysis of protein expression patterns and physiological analysis revealed that proteins associated with changes in energy usage; osmotic adjustment; ROS scavenging; and protein synthesis, processing, and proteolysis play important roles in maintaining root growth under drought stress. This study provides new insight for better understanding of the molecular basis of drought stress responses, aiming to improve plant drought tolerance for enhanced yield.

## 1. Introduction

Plants encounter a variety of abiotic stresses during the growth period. These stresses unbalance cellular homeostasis and lead to morphological, physiological, and molecular changes [1]. These changes have a negative impact on survival, biomass production, and grain yield [2]. Global warming and climate change may also be exacerbating the effects of abiotic stresses on crop production in many areas of the world. Drought in particular severely impairs plant growth and development and limits crop productivity more than any other environmental factors [3,4]. As the climate continues to change, drought may become more frequent and can cause severe problems [5].

As water resources for agriculture are becoming increasingly limited, the development of enhanced crop tolerance to water restriction conditions would be the most economical approach to improving agricultural productivity. Thus, understanding plant’s responses to drought and breeding plants for increased drought tolerance are two major goals of plant biologists and crop breeders.

Water is an essential solvent for cell biochemical reactions and is indispensable for life. Extreme dehydration reduces cell turgor and adversely affects cellular metabolic processes. A reduced water availability induces many changes at the morphological, physiological, biochemical, and metabolic levels in all plant organs. There is hardly a physiological process in plants that is not impaired by water deficit [6]. Prolonged water deficits, such as that imposed by severe droughts, result in leaf wilting and ultimately end in plant death.

Drought stress responses in plants occur at various organ levels, among which the root-specific processes are of particular importance. Under normal growth condition, roots absorb water and nutrients from the soil and supply those throughout the plant body, thereby playing pivotal roles in maintaining cellular homeostasis. However, this balanced system is altered during the stress period when roots are forced to adopt several structural and functional modifications. Examples of these modifications include molecular, cellular, and physiological changes such as alteration of metabolism and membrane characteristics, accumulation of compatible solutes like proline, induced oxidative stress, and reduction of root length [7,8]. Plants generate reactive oxygen species (ROS) under oxidative stress conditions. ROS are key secondary messengers triggering subsequent defensive measures in plants [9,10]. Plants developed a sophisticated and elaborate system for scavenging high levels of ROS using antioxidant enzymes that include superoxide dismutase (SOD), catalase (CAT), peroxidase (POD), and ascorbate peroxidase (APX). Drought stress produces changes in the expression of a related set of genes, inhibiting the synthesis of normal proteins and resulting in the production of stress-specific proteins [1]. To mitigate and recover from the damaging effects of water deficit from environmental conditions, plants have evolved various adaptive strategies at the cellular and metabolic levels. Most of these strategies involve dynamic changes in protein abundance. These changes are often caused by drought stress responsive pathways that can be best explored at the global level using high-throughput approaches such as proteomics [11]. Studying the differentially expressed proteins in the proteome of roots under drought stress will provide a better understanding of the drought-responsive mechanisms in plant seedlings. Also, elucidating the mechanisms of drought resistance in plants adapted to arid environments may provide new strategies for engineering drought tolerance in crop species [12].

Sorghum (*Sorghum bicolor* (L.) Moench) is the fifth most important grain crop in the world and has been bred for grain, sweet stems, high-energy fiber, and other multi-purpose uses [13]. It is grown widely in semiarid and tropical regions of Africa and Asia due to its drought tolerance. Sorghum usually does not compete with crops such as maize, rice, and wheat for land area, as it grows where these crops are not adapted, making it an ideal food security crop in these regions [14]. Sorghum can tolerate drought, high salinity, and other abiotic and biotic stresses [13]. Sorghum can become dormant under adverse conditions and can resume growth when the environment is more favorable, which contributes to its drought tolerance [15]. Sorghum has been exploited for soil and water conservation for many years. However, the drought-resistance molecular mechanisms possessed by this species remain unclear.

Proteomics is a powerful tool for analyzing the functions of plant genes or proteins [16]. Proteomics allows qualitative and quantitative measurements of proteomes in specific plant tissue at specific developmental and physiological stages [17]. As a technique, proteomics is advantaged over other “omics” tools since proteins are the key players in the majorities of cellular events [18]. Proteomics is the best available molecular tool for understanding the complex molecular mechanism in response to drought stress [19,20,21,22] and has been applied to research on drought stress in rice, maize, wheat, barley, peanut, and so on [12,23,24,25,26,27,28,29,30].

Sorghum has a small genome (730 Mb), making it a good species for genomic and proteomic research [13]. The availability of the sorghum whole genome sequence from an inbred line BTx623 placed 98% of its genes in their chromosomal context [31]. The combination of the natural drought tolerance traits of sorghum and the genome sequencing milestone of sorghum [31] makes it a model plant for proteomics and genomics research. Considering the importance of sorghum stress resistance, its resistance molecular mechanism research is of broad interest. Recently, Abdel-Ghany et al. (2020) performed a transcriptome analysis in seedlings of sorghum genotypes by Polyethylene Glycol (PEG)-induced drought stress to identify the changes in gene expression that are unique to drought-resistant genotypes of sorghum and revealed a set of drought-regulated genes, including many genes encoding uncharacterized proteins that are associated with drought tolerance at the seedling stage [32]. This study reflects the molecular characteristics of sorghum resistance/tolerance to drought stress and give us useful transcriptome study data of sorghum response to water deficit. The first reported sorghum proteomics study profiled 2-DE protein patterns of the total soluble proteins and secreted culture filtrate protein in a cell culture. This leads to comprehensive mapping and characterization of the sorghum cell suspension culture secretome [33]. This was followed by a study using a sorghum cell suspension treated with sorbitol to induce drought stress and to obtain fractions enriched for sorghum extracellular matrix (ECM) proteins, and they found a significant increase of ECM proteins under drought stress conditions in cell culture. They concluded that protein secretion is a major response of sorghum to osmotic stress [34]. Another study of sorghum also used physiological and comparative proteomics to study abiotic stress response and to identify protein groups responding to drought, salinity, and Cd/Cu stress in sorghum [35,36,37,38,39,40,41].

The majority of studies that have investigated abiotic stress response in sorghum have focused on the leaves; these results suggest the important contribution of sorghum leaf proteome in coping with stress. However, until now, the comprehensive proteomic evaluation of drought-stressed roots of sorghum has been very limited. Thus, elucidating the molecular mechanisms of the sorghum root response to drought stress is critical.

Considering the lack of information about sorghum root response to drought stress at the protein level and because of the importance of roots in drought tolerance, a proteomics technique was used in this study to compare changes of proteins induced by drought stress in roots of sorghum seedling to provide information about root function in drought tolerance at the proteome level.

Roots are the organs where plants first encounter drought stress and may be able to sense and respond to this stress condition. Therefore, we carried out a proteomic and physiological analysis of sorghum roots under PEG-imitation drought stress at the seedling stage.

Therefore, in this study, we present a comprehensive physiological and comparative proteomic analysis of drought-tolerant sorghum seedling roots under 10% PEG-6000 stress using a plant physiology method, 2-D combined with mass spectrometry proteomics, and bioinformatics analysis.

## 2. Results

### 2.1. Effect of Drought Stress on MDA and Proline Contents in Roots

**Figure 1:**
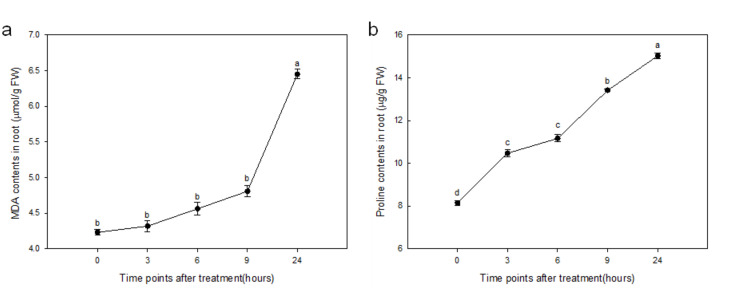
Changes in malondialdehyde (MDA) (**a**) and proline (**b**) contents of sorghum root in response to Polyethylene Glycol (PEG) simulated drought stress (two-week-old seedlings treated with 10% PEG-6000). Data are presented as mean ± SD (*n* = 3). Different letters indicate statistically significant differences at *p* < 0.05.

We determined the malondialdehyde (MDA) and proline contents in the root of sorghum at different time points after drought stress treatment. Before drought stress, MDA and proline contents were 4.23 µmol/g FW and 8.15 µg/g FW, respectively. During drought stress, contents of MDA and proline were increased gradually and reached 6.45 µmol/g FW and 15.02 µg/g FW, respectively, after 24 h of treatment (Figure 1).

### 2.2. Drought Stress Increased Antioxidant Enzyme Activity in Roots

**Figure 2:**
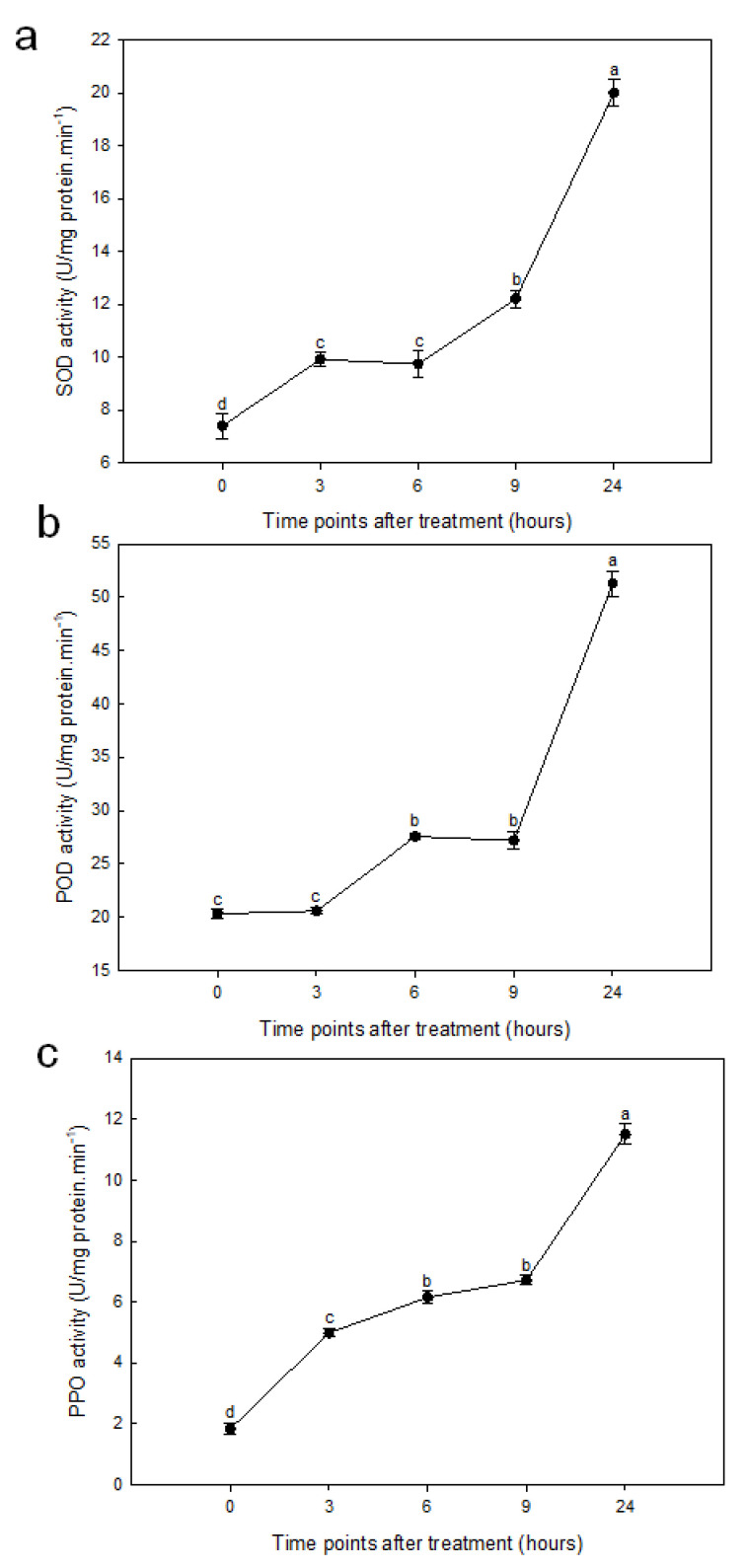
Antioxidative enzyme (**a**) superoxide dismutase (SOD); (**b**) peroxidase (POD); and (**c**) polyphenol oxidase (PPO) activity of sorghum root in response to PEG simulated drought stress (two-week-old seedlings treated with 10% PEG-6000). Data are presented as mean ± SD (*n* = 3). Different letters indicate statistically significant differences at *p* < 0.05.

The activities of SOD, POD, and polyphenol oxidase (PPO) were increased gradually from 0–9 h posttreatment, followed by significant increase from 9 h to 24 h posttreatment (Figure 2).

### 2.3. Changes in Proteomic Expression Patterns of Roots in Response to Imitation Drought Stress

**Figure 3:**
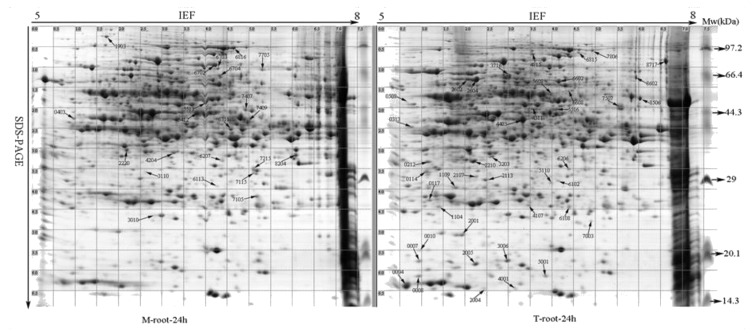
Two-dimensional gel analysis with proteins isolated from root of normal sorghum seedling (M-root-24 h) or root of PEG-6000 treated sorghum seedlings (T-root-24 h) and harvested at 24 h posttreatment: the arrows point to protein spots (automatic allocation of protein serial number by PDQuest Software) with altered expression levels (fold change > 2.0, quality score > 80, and *p* < 0.05) and were selected for MALDI-TOF-TOF MS analysis.

There was a broad distribution of the proteins in terms of pI (5.0–8.0) and mass (10–100 kDa) (Figure 3). Approximately 906 protein spots were reproducibly detected, of which 412 spots did not match between different replications or were only found in the mock inoculation. Image analysis revealed 494 common reproducible protein spots across all samples. Among them, 105 exhibited significant changes to spot abundance (*p* < 0.05) (Figure 3). Sixty protein spots (12.1%) were upregulated and 45 (9.1%) were downregulated in the PEG-treated plants compared to the no PEG treatment control (Table 1). Among those differentially expressed protein spots, there were 43 upregulated spots (Table 2) and 22 downregulated spots (Table 3) that had 2.0 fold change or greater change compared to the control and a quality score of over 80.

### 2.4. Identification of Drought-Responsive Proteins by MALDI-TOF-TOF MS Analysis

When subjected to MALDI-TOF-TOF mass spectrometry analysis of the selected spots and subsequent Mascot search by Peptide mass fingerprinting (PMF), 52 of the 65 spots were identified successfully by MALDI-TOF-TOF MS; the detailed information of the identified protein spots in the present work are summarized in Table 4.

### 2.5. Functional Classification and Subcellular Localization of Identified Proteins

To understand better the traits possessed by drought tolerant plants and the roles of the proteins involved in drought stress responses, the functional classification of identified proteins was carried out using gene ontology (http://geneontology.org/) based on the sorghum and maize genome annotation project database. Protein subcellular location was identified using SIB Bioinformatic resource portal (http://www.expasy.org/proteomics) with UniProtKB Complete proteome (http://www.uniprot.org/) annotation project databases. Gene ontology categories were assigned to all 52 protein spots according to their biological processes and cellular component localization (Table 5).

**Figure 4:**
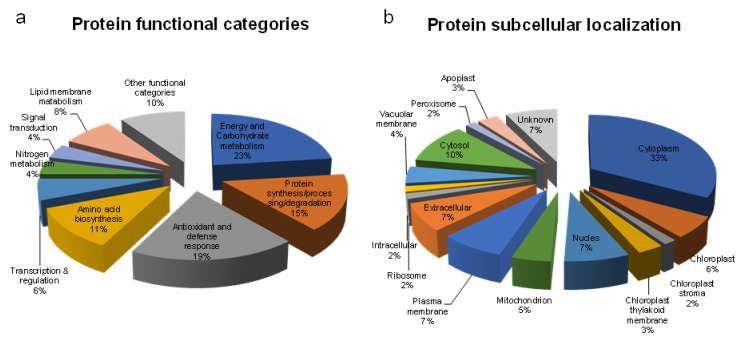
The frequency distribution for the 52 identified proteins in the root of sorghum seedlings within functional categories determined based on their biological functions (**a**) and subcellular localization (**b**).

According to their biological function, the identified proteins were classified into the following 9 categories: carbohydrate and energy metabolism, antioxidant and defense response proteins, protein synthesis/processing/degradation, amino acid biosynthesis and nitrogen metabolism, transcription and regulation, signal transduction, lipid membrane metabolic, and other functional categories. However, among all the identified proteins, most of the proteins were found to be involved in carbohydrate and energy metabolism, followed by antioxidant and defense response and protein synthesis/processing/degradation (Figure 4a).

Based on the subcellular localization, the identified proteins were grouped into 15 categories (Figure 4b). Most of the proteins were localized into the cytoplasm and cytosol, followed by plasma membranes, nucleus and extracellular, chloroplast, vacuolar membrane and mitochondrion, chloroplast thylakoid membrane and apoplast, chloroplast stroma, ribosome, peroxisome, intracellular, and uncharacterized (Figure 4b).

## 3. Discussion

### 3.1. Physiological Alteration in Roots of Sorghum in Response to Drought Stress

The roots of sorghum seedlings exhibited a different accumulated change of biometric parameters when exposed to imitation drought stress. In the early stage of PEG-imitation stress, PEG-6000 mainly induced osmotic stress, which resulted in the accumulation of osmotic-protectants such as proline. It has been described as a primary defense response of a plant to maintain the osmotic pressure in the cell [42,43]. The capacity to accumulate proline under stress conditions has been correlated with stress tolerance in several plant species [44,45]. Proline is a cytoplasmic osmolyte that contributes as a source of carbon and nitrogen during post stress recovery and growth [46]. Recently, it was reported that stress-inducible proline accumulation might therefore act as a component of an antioxidative defense system to counteract the deleterious effects of oxidative stress by directly scavenging free radicals or by activating antioxidant systems [47]. Similar trends in higher accumulation of proline in drought-tolerant sorghum varieties during periods of water limitation have also been reported [35,36]; these are consistent with our results. The elevated level of proline found in drought-treated roots indicates that sorghum had the ability to regulate drought stress tolerance. The increase of MDA level indicates oxidative stress injury and/or cellular damage due to ROS production. Drought-induced lipid peroxidation was also found in soybean roots [21], which is in agreement with the results of this study. Similarly, Deeba et al. (2012) observed a significant increase of MDA in cotton leaves in response to drought stress, indicating that MDA was increased under drought stress [48], which suggested that the whole regulatory network in plants may alter with a series of events. The levels of proline and MDA have similar variation trends in roots with that in leaves of sorghum during stress. The contents of proline and MDA in roots reached a relatively lower level (6.45 µmol/g FW and 15.02 µg/g FW, respectively) at 24 h posttreatment compared to 23.59 µmol/g FW and 375 µg/g FW, respectively, in leaves (data not shown) (Figure 1), which indicated that the difference of accumulation of ROS between leaves and roots at the early stress stage implies that the oxidative stress injury and/or cellular damage in roots is much smaller than that in leaves.

Analysis of the antioxidant enzymes revealed that the activities of SOD, POD, and PPO have similar variation trends in sorghum roots in the duration of the stress. Plants have an internal protective enzyme-catalyzed clean up system, which is fine and elaborate enough to avoid injuries of ROS, thus guaranteeing normal cellular function [49]. The balance between ROS production and activities of an antioxidative enzyme determines whether oxidative signaling and/or damage will occur [50]. Maintaining a high level of antioxidative enzyme activities may contribute to drought induction by improving the ability against oxidative damage [51]. Our results indicated that the activities of SOD, POD, and PPO in roots increased gradually with the duration of stress in the early stage of treatment. The activities of SOD and POD in roots are obviously higher than that in leaves at 24 h posttreatment, which reached 20.01 and 51.32 U/mg protein/min (Figure 2), respectively, while in leaves, it achieved 8.83 and 4.11 U/mg protein/min, respectively (data not shown); the activity of PPO in roots is similar to that in leaves. Stronger SOD and POD activities are beneficial to the removal of ROS; this is also one of the reasons that oxidative damage in roots is less than that in leaves, confirmed by lower MDA levels, which proved that efficient antioxidative characteristics can provide better protection against oxidative stress in roots under imitation drought stress.

Physiological analysis indicates that the defense response induced by drought stress were triggered by 24 h posttreatment. Therefore, sampling at 24 h posttreatment was the suitable time point for proteomic analysis. To increase the drought tolerance of crop species, we need to understand better the traits possessed by drought tolerant sorghum plants. The present experiments were carried out to investigate the molecular mechanism and, more precisely, the alterations in the proteome induced in sorghum roots under imitation drought stress. These proteomic data, in combination with physiological analyses, provide insights into the underlying mechanisms of drought tolerance in hydroponically grown sorghum plants.

### 3.2. Proteins Involved in Energy and Carbohydrate Metabolism

Carbohydrate and energy metabolism are essential for root development and stress response, which is thought to be the most critical pathways in plants that regulate sugar synthesis and transformation as well as carbon partitioning [41]. In this study, energy and carbohydrate metabolism were significantly altered in response to drought stress, and a total of eight protein spots involved in energy and carbohydrate metabolism were found to be upregulated by drought stress, including uncharacterized protein (spots 2602, 2604, 3203, 3716, and 6608), sucrose synthase (EC = 2.4.1.13) (spot 4815), and pyrophosphate–fructose 6-phosphate 1-phosphotransferase subunit beta (spots 6206, 6602). Uncharacterized protein (spots 2602 and 2604) is proposed to participate in glucose catabolic process. Protein spot 3203 is related to the glyoxylate cycle. Spot 3716 was thiamine pyrophosphate and has carboxylyase activity, which is involved in the tricarboxylic acid (TCA) cycle. Under drought stress, the accumulation of carbohydrates such as sugars (glucose, fructose, sucrose, and fructan) and starch play important roles in osmotic adjustment and carbon storage [52]. Sucrose synthase (spot 4815) plays a very important role in sugar metabolism; it is closely related to the process of sugar decomposition and energy supply. Proteome studies in maize and rice roots reveal that this enzyme increases its expression levels in response to salinity [53] and drought [54]. While it has been reported that this protein decreased its level in sorghum root during water limitation [36], this may be due to differences in stress duration and intensity. Pyrophosphate-fructose 6-phosphate 1-phosphotransferase subunit beta (PFP) is a key enzyme of glycolysis process; PFP catalyzes the interconversion between fructose-6-phosphate and fructose-1, 6-bisphosphate. This process is catalyzed by phosphofructokinase (PFK) in most cases, and the difference is that the reaction catalyzed by PFK is irreversible and requires ATP, while the reaction catalyzed by PFP is reversible and ATP is replaced by pyrophosphoric acid. Studies have shown that the effect of PFP can enhance the rate of glycolysis and the production of ATP under hypoxic conditions [55]. In our study, PFP was significantly upregulated in roots of sorghum seedling. Fructose 6-phosphate 1-phosphotransferase has also been found increase in maize roots [12] under drought treatment. The increase in PFP expression may be an adaptive mechanism for sorghum roots to cope with drought stress through glycolysis. During drought stress in roots of sorghum seedling, the increased abundance of sucrose synthase (spot 4815), PFP (spots 6206 and 6602), and uncharacterized proteins (spots 2602, 2604, 3203, 3716, and 6608) could be related to the cellular requirement for extra energy in order to deal with water deficit and to repair damage. Enhanced levels of these primary metabolism-related enzymes thus indicate that adequate energy is a prerequisite for roots to deal with drought.

Four protein spots associated with carbohydrate and energy metabolism showed low abundance under drought stress, which were identified as phosphoglycerate kinase (EC = 2.7.2.3) (spot 0403), PsbP domain-containing protein (spot 3010), 6-phosphogluconate dehydrogenase (EC = 1.1.1.44) (spot 5513), and RmlD_sub_bind domain-containing protein (spot 6207). Phosphoglycerate kinase (PGK) is involved in gluconeogenesis and glycolytic process, which has been identified in the roots of rice [56], wheat [57], tomato [58], and creeping bent grass [59] under salt stress. However, its expression patterns vary among different plant species and salt treatment conditions; this protein increased their abundance in rice and wheat root under salt drought while decreased the expression level in the roots of tomato and creeping bent grass, which is consistent with our results. PsbP domain-containing protein (spot 3010) is an enzyme related to the Calvin cycle. Some chloroplast-related proteins that were expressed in the roots may have been transported from chloroplasts or produced in pro-plastids. Moderate reduction of the photosynthesis-related enzymes has been detected in roots of sorghum and rice under drought stress [39,54], implying that the decrease of the enzymes associated with carbon fixation under drought stress were general adaptation syndromes. 6-phosphogluconate dehydrogenase (spot 5513) is related to pentose phosphate pathway (PPP); PPP generates NADPH for reductive biosynthesis reactions and carbon skeletons for the synthesis of nucleotides, aromatic amino acids, phenylpropanoids and their derivatives. It has been reported that several PPP-related proteins (including 6-phosphogluconate dehydrogenases) decreased their expression levels in the roots of Arabidopsis [60] and barley [61] under salinity. RmlD_sub_bind domain-containing protein (spot 6207) is responsible for UDP-rhamnose and dTDP-rhamnose biosynthesis. Downregulation of the above proteins suggested that the biosynthesis of carbohydrate was inhibited under drought conditions.

### 3.3. Proteins Involved in Antioxidant and Defense Response

Under stress condition, excess ROS is produced, which is removed by ROS scavengers [18]. Several antioxidant proteins were identified in this study, such as SOD (spot 0008), POD (spot 0114), and CAT (spot 8506), which were expressed more under water deficit conditions (Table 2). Adverse stress, such as drought, usually results in excessive accumulation of ROS in plant cells, which eventually leads to oxidative stress. SOD, POD, and CAT are important antioxidant enzymes for organisms and act synergistically to eliminate harmful free radicals under adverse conditions in a variety of crops [18,60,62,63,64,65,66]. Our results demonstrated that sorghum seedling roots also possess a ROS scavenging system that responds to drought stress. Drought stress induced accumulation of more antioxidant enzymes, which can more effectively eliminate ROS. This is consistent with the physiological results of SOD and POD. In addition, a higher abundance of ROS scavengers was detected in roots under drought stress and can be looked upon as a preventive measure against oxidative damage caused due to high ROS levels. Two enzymes involved in aldehyde metabolism in sorghum roots were upregulated under drought stress, including uncharacterized protein (spot 4403) and aldehyde dehydrogenase (spot 5608). ROS can promote the peroxidation of membrane lipids, leading to the accumulation of aldehydes in the organism and damage to the cells [16]. Uncharacterized protein (spot 4403) is related to a formaldehyde catabolic process, which can convert toxic formaldehyde into a nontoxic substance. Mitochondrial aldehyde dehydrogenase (spot 5608) can catalyze the oxidative dehydrogenation of endogenous or exogenous aldehydes to generate corresponding carboxylic acids by combining NAD^+^ or NADP^+^ to reduce the accumulation of aldehydes in plants and to achieve detoxification [67]. Studies have found that aldehyde dehydrogenase is upregulated to reduce the toxicity of aldehydes under drought stress [68]. In addition, LEA-like protein (spot 8602) and uncharacterized protein (spot 6108) increased their expression levels in sorghum roots under drought stress, uncharacterized protein (spot 6108) is a stress-related protein, and stress-related proteins have been observed to play pivotal roles in plant resistance to environment stress [69,70,71]. LEA-like proteins are hydrophilic proteins induced by drought stress, which are involved in plant protection from dehydration-associated injury [72]. This protein has been proposed to contribute to membrane and protein stability, metal scavenging, and suppression of ROS-induced damage that occurs in plants exposed to water deficit [32,73,74,75]. These findings together indicate that the abovementioned enzymes play key roles in protecting root cells from drought-induced oxidative damage.

In the present study, three protein spots including uncharacterized protein (spot 6702), Pyr_redox_2 domain-containing protein (spot 6703), and Aldo_ket_red domain-containing protein (spot 8204) were expressed at lower levels in drought-treated sorghum seedling roots. Uncharacterized protein (spot 6702) is a Hsp90 protein binding protein which belongs to the class of heat shock protein and function as molecular chaperone. Proteomic studies have shown that Hsp90 levels were decreased in the roots of *Agrostis stolonifera* [59] and maize [53] under salt conditions. Pyr_redox_2 domain-containing protein is a stress response protein and related to oxidation-reduction process. Aldo_ket_red domain-containing protein is responsible for siderophore biosynthetic process. It has been shown that this protein responds to iron starvation. The decreased abundance of these three proteins might indicate that protein synthesis, processing, and turnover are impaired under drought stress.

### 3.4. Proteins Involved in Protein Synthesis/Processing/Degradation

Four upregulated protein spots, eukaryotic translation initiation factor 5A (eIF-5A, spots 2005 and 3006), small ribosomal protein 4 (rps4, spot 7507), and uncharacterized protein (spot 0212), are responsible for efficient protein synthesis and transport. Protein synthesis plays a pivotal role in abiotic stress adaptation. Proteomics studies have revealed many components of protein synthesis machinery to be altered in expression under drought stress conditions, including different ribosomal proteins, translation initiation factors, translation elongation factors, and chaperone [66]. Small ribosomal proteins (RPs) are related to ribosome assemble and protein translation and form part of the ribosomal stalk, playing a crucial role in the interaction of the ribosome with GTP-bound translation factors. RPs are essential for protein synthesis and have been revealed to play an important role in metabolism, cell division, and growth [76]. Increased abundance of these proteins has been reported in the leaves of sorghum under salt/drought and Cd/Cu stress [38,39,40,41], in sugarcane, and in rice under salt/drought stress [69,70,77]. Increased levels of protein involved in synthesis and transport are therefore important in the plant cell’s metabolic activities and general growth.

Four the four downregulated protein spots including UBA_e1_C domain-containing protein (spot 1903), probable protein phosphatase 2C 76 isoform X2 (spot 3110), eukaryotic translation initiation factor 3 subunit I (eIF3i, spot 6311), and aspartate–tRNA ligase 2 cytoplasmic (spot 6704), UBA_e1_C domain-containing protein (spot 1903) is involved in ubiquitin-dependent protein catabolic process; probable protein phosphatase 2C 76 isoform X2 (spot 3110) has protein serine/threonine phosphatase activity, which is related to protein posttranslation modification; and Eukaryotic translation initiation factor 3 subunit I (eIF3i, spot 6311) and aspartate–tRNA ligase 2 cytoplasmic (spot 6704) are responsible for protein biosynthesis. It has been reported that the expression of most of the protein synthesis-related genes was reduced in Arabidopsis roots under salt stress [60]. In addition, several eukaryotic translation initiation factors (eIFs) including eIF3, eIF5A, eIF5A3, and eIF4A3 were NaCl-reduced in roots from Arabidopsis [60], rice [78], wheat [57,62], and sugar beet [79]. The decreased abundance of these five proteins might indicate that protein synthesis, processing, and turnover are suppressed under drought stress.

The differential regulation of various components of the translation machinery implies that a complicated mechanism governing protein synthesis/processing and proteolysis exists in response to drought stress.

### 3.5. Proteins Involved in Transcriptional and Regulation

Transcriptional regulation of drought-responsive genes is an important strategy for plants to respond to various stress conditions [60]. Proteomics studies have revealed that the levels of transcription-related proteins are responsive to drought stress and play a pivotal role in drought tolerance [69]. Regulatory proteins, including N-Acety-L-Cysteine (NAC) domain-containing protein (spot1109) and Ribonuclease (spot 4001) were markedly upregulated by drought stress in this investigation. NAC domain-containing protein (spot 1109) is a kind of transcription factor and is supposed to regulation of transcription. A previous report has indicated that NAC domain-containing protein was significantly induced in the roots of Arabidopsis [60] by salt stress and in the roots of sorghum by water deficit [36]. Ribonuclease (spot 4001) belongs to an RNA processing enzyme which functions in posttranscriptional gene silencing by RNA. Ribonuclease for RNA degradation has been found to increase its abundance in the roots of wheat in response to salt stress [57]. These proteins are important regulatory components in the transcriptional networks and may control diverse processes to cope with drought stress.

In the present study, heterogeneous nuclear ribonucleoprotein 1 (spot 7407) was decreased by exposure to water deficit (Table 3). This protein has been reported to increase its level in the leaves of Arabidopsis under salinity [80]; these differences in expression may be due to differences in organs and stress types. Downregulation of this protein might indicate more efficient RNA transcription under water deficit conditions.

### 3.6. Proteins Involved in Amino Acid Biosynthesis and Nitrogen Metabolism

During drought stress, proteins are involved in amino acid metabolism, except MR_MLE domain-containing protein (spot 0313), which was increased under water deficit. Other proteins including cysteine synthase (spots 2220 and 4204), DUF3700 domain-containing protein (spot 6113), glutamate dehydrogenase (spot 7409), and uncharacterized protein (spot 7705) were found to be downregulated in sorghum roots. Cysteine synthase is a crucial enzyme related to the synthesis of cysteine and is known to increase the abundance of glutathione (GSH); GSH is a key component of the GSH-ascorbate cycle, which can reduce toxic hydrogen peroxide [81]. During oxidative and salt stress, cysteine synthase decreased its abundance in the shoot of wheat seedling [82] and the leaves of *Puccinellia tenuiflora* [83]. Glutamate decarboxylase (GAD) is a Ca^2+^-dependent CaM binding protein that catalyzes the synthesis of γ-aminobutyrate (GABA). GABA is involved in several physiological processes, such as nitrogen metabolism, cytosolic pH regulation, and carbon flux into the TCA cycle [64]. It has been reported that GAD abundance was decreased in salt-stressed wheat roots [62], implying the inhibition of GABA synthesis in roots under abiotic stress. DUF3700 domain-containing protein is involved in glutamine metabolic process. Uncharacterized protein (spot 7705) is proposed to be related to the formyltetrahydrofolate biosynthetic process. The decreased abundance of these five protein spots might indicate that the biosynthesis of amino acids in the roots of sorghum seedling was markedly suppressed under water deficit.

Two protein spots involved in nitrogen metabolism enhanced their abundance under drought stress, including Rieske-like (2Fe-2S) domain containing protein (spot 5001) and glutamine amidotransferase type-2 domain-containing protein (spot 6102). Glutamine amidotransferase type-2 domain-containing protein is related to ammonia assimilation and the glutamate biosynthetic process. Rieske-like (2Fe-2S) domain containing protein has nitrite reductase activity and is involved in nitrate assimilation; nitrite reductase is reported to play a pivotal role in nitrate assimilation and is a key enzyme in the natural nitrogen cycle; and nitrate accumulation can be induced in plants subjected to water deficiency [70]. Desiccation results in cytoplasmic increases in concentrations of toxic ions, such as NO_3_^−^, which may inhibit metabolic processes, and excessive nitrate are toxic to plants. Nitrite reductase can degrade nitrite to NO or NH_3_, thus reducing the toxicity and providing precursors for nitrogenous metabolites; therefore, it is critical for plant growth and drought stress response. Upregulation expression of this protein has been reported in the leaves and roots of wheat under salt stress [57,62]. In the present study, overexpression of nitrite reductase homologous and ammonia assimilation-related proteins in the roots of sorghum indicate that these enzymes play important roles to cope with water deficit.

### 3.7. Proteins Involved in Lipid Membrane Metabolic

Two protein spots associated with lipid metabolism, including patatin (spot 5516) and lipoxygenase (spot 7806) were found to be enhanced their abundance under drought stress (Table 2). Patatin is associated with lipid degradation, which provides energy by degrading lipids. Upregulated patatin may meet the energy requirements of the root during drought stress. Lipoxygenase is proposed to be responsible for lipid biosynthesis and fatty acid metabolism. Lipid synthesis and efficient transport are important to maintain cell structure homeostasis under stress conditions [69]. Proteomic studies have found that lipoxygenase is upregulated in the roots of barley under salt stress [61]. However, for the other two proteins, the expression of epimerase domain-containing protein (spot 5412) and prohibitin (spot 7115) was decreased in the roots of sorghum seedling under drought stress. Epimerase domain-containing protein is responsible for steroid biosynthetic process. It has been reported that the membrane steroid binding proteins were identified as salt-responsive proteins in the roots of rice, pea, tomato, and sugar beet [18,62,64]. The mitochondrial prohibitin complex is localized in the mitochondrial inner membrane. This complex is involved in the stabilization of newly synthesized mitochondrial-encoded proteins, which play a crucial role in mitochondrial biogenesis and protection against stress and senescence in plant cells [84]. Decrease of prohibitin indicates that the protein complexes were impaired under water deficit. Fatty acids and steroid constitute major components of membrane lipids. Water deficit can lead to alteration of fatty acid and steroid composition in plasma membrane of root, which may be required for maintaining membrane function in order to cope with drought stress. Therefore, the differential expression of these four protein spots suggest that drought may alter lipid metabolic; thus, components of the membrane are rapidly remodeled to allow cell adjustment for drought tolerance.

### 3.8. Proteins Involved in Signal Transduction

In the present study, Bet_v_1 domain-containing protein (spot 0004) and uncharacterized protein (spot 1104) were identified to be upregulated by drought stress. Bet_v_1 domain-containing protein was related to defense response and participated in abscisic acid (ABA)-activated signaling pathway; uncharacterized protein (spot 1104) was involved in phosphorelay signal transduction system. Upregulated, these two proteins indicate that the signal transduction pathways triggered by drought have been activated.

### 3.9. Other Functional Categories and Uncharacterized Protein

In this study, four upregulated protein spots in response to drought stress were identified as NAB domain-containing protein (spot 2107) and uncharacterized protein (spots 2210, 4511, and 8717). NAB domain-containing protein was related to actin binding; uncharacterized protein (spots 4511) was involved in the regulation of ABA biosynthetic process. The function of other two proteins remain unknown. One downregulated protein spot (7215) was identified as Dirigent protein, which was involved in plant secondary metabolism, while the accurate function remains unclear. As a C_4_ cereal, although the entire genome sequence of sorghum has been available since 2009 [34], most sorghum gene products remain experimentally uncharacterized and are submitted as hypothetical proteins in protein database. Hypothetical proteins are proteins predicted from genome sequences but for which the existence has not been experimentally proven at the protein level [41]. However, the present results are consistent with other findings that have been reported recently for sorghum [33,34,35,36,37,38,39,40,41].

The 52 positively identified proteins represented 49 unique gene products owing to the appearance of similar proteins in multiple spots on the 2-DE gel, differing in Mr, pI, or both, such as eukaryotic translation initiation factor 5A (2005 and 3006), pyrophosphate--fructose 6-phosphate 1-phosphotransferase subunit beta (6206 and 6602), and uncharacterized protein (2602 and 2604). Glycosylation, phosphorylation, and other posttranslational modifications (PTMs) which can alter the molecular weight and/or charge of proteins may be responsible for these results. Such detected protein spot patterns have been described in many previously studies [33,34,38,64,69,83,85]. Multiple spotting of the same protein in different locations on a 2-DE gel may be reflections of protein isoforms due to PTMs or multigene families, products of proteolytic activities, translation of alternatively spliced mRNAs, the attendance of multiple subunits of a single protein, and/or the chemical modification of proteins during sample preparation.

In addition to previously reported drought stress responsive proteins, we identified several novel proteins such as the NAB domain-containing protein (spot 2107), Dirigent (spot 7215), DUF3700 domain-containing protein (spot 6113), Bet_v_1 domain-containing protein (spot 0004), epimerase domain-containing protein (spot 5412), prohibitin (spot 7115), RmlD_sub_bind domain-containing protein (spot 6207), and some uncharacterized protein (spots 2210, 2602, 4511, and 8717) in sorghum roots. The exact function of these proteins in combating drought is unclear. Future functional studies with the uncharacterized proteins will further our understanding of drought tolerance in sorghum at the seedling stage and help in developing drought-resistant crops.

## 4. Materials and Methods

### 4.1. Seedling Cultivation, Polyethylene Glycol (PEG) Treatment

Surface-sterilized seeds of the sorghum (*Sorghum bicolor*) inbred line BTx623 (which is the genotype for sequencing the sorghum genome, http://www.phytozome.net/sorghum) were germinated in an incubator at 25 °C for 4 days. After germination, healthy seedlings were transplanted into a plastic container with 4 L of half strength Hoagland culture solution. After 6 days of transplanting, the culture solution was changed to full strength. The culture solution was continuously aerated, and the pH was adjusted to 6.0 with 0.1 M HCl or 1 M KOH daily. Twelve days after transplanting, 10% PEG-6000 (−0.2 MPa) was imposed at 08:00 AM to induce drought stress, and unless stated otherwise, root tissues samples were harvested and measurements were made after 0, 3, 6, 9, and 24 h of PEG treatment. Half plants were not treated with PEG as controls. For physiological analysis, each treatment includes three replications and each replication includes two technical replications. For proteomics analysis, three biological replicates were performed.

### 4.2. MDA Contents Measurement

Lipid peroxidation was estimated by measuring MDA contents according to a modified thiobarbituricacid (TBA) method [86]. Approximately 0.1 g of root fresh tissues was ground in 1.0 mL of 10% trichloroacetic acid (TCA) using a mortar and pestle. The homogenate was centrifuged at 10,000 rpm for 20 min. The reaction mixture containing 0.5 mL of extract and 1 mL of TBA was heated at 100 °C for 30 min, quickly cooled on ice, and then centrifuged again at 10,000 rpm for 20 min. The absorbances at 450, 532, and 600 nm were determined using an ultraviolet spectrophotometer (UV-2550, Shimadzu, Kyoto, Japan).

### 4.3. Antioxidative Enzyme Activity Assays

For analysis of the activity of SOD, POD, and PPO, total soluble protein was extracted from the root samples. Frozen root tissues (approximately 0.1 g) were homogenized in ice-cold 50 mM potassium phosphate buffer (pH 7.0) containing 1 mM EDTA-Na_2_ and 2% (*w*/*v*) polyvinylpolypyr-rolidone (PVPP) using a precooled mortar and pestle. The homogenate was centrifuged at 15,000× *g* for 20 min at 4 °C, and the supernatant was used immediately for enzyme assays. Protein concentration was determined according to the Bradford (1976) method using Bio-Rad protein assay kit and bovine serum albumin as a standard [87]. SOD (EC 1.15.1.1) activity was estimated based on its ability to inhibit the photochemical reduction of nitro blue tetrazolium (NBT) at 560 nm [88]. The POD activity was assayed according to the method described by Kwak et al. (1995) using pyrogallol as a substrate [89]. One unit of POD activity was defined as the amount of enzyme required to form 1 mg of purpurogallin from pyrogallol in 20 s, as measured by absorbance at 420 nm. The PPO activity was determined by measuring the increase in absorbance at 420 nm every 15 s for up to 5 min. The reaction mixture contained 0.05 mL of enzyme solution, 0.4 mL of 100 mM catechol, and 0.8 mL of 0.1 M phosphate buffer with a PH of 5.0 at 25 °C. The reaction mixture without the enzyme extract served as a control, and the buffer was used as blank. Enzyme activity was calculated from the linear portion of the curve. One unit of PPO activity was defined as 0.01 of absorbance increase per min.

### 4.4. Measurement of Free Proline Content

The free proline content of drought-treated plants was measured using a spectrophotometer according to the method of Bates et al. [86]. Leaf tissue (0.1 g) was homogenized in 3 mL of sulfosalicylic acid (3%) and centrifuged at 10,000× *g* for 15 min. Then, 1 mL of the supernatant was added to a test tube, along with 1 mL glacial acetic acid and 1 mL ninhydrin reagent. The reaction mixture was boiled in a water bath at 100 °C for 30 min. After cooling, 2 mL toluene was added to the reaction mixture, which was then vortexed for 30 s. The upper phase (containing proline) was measured with a spectrophotometer (UV-2550, Shimadzu, Kyoto, Japan) at 520 nm using toluene as the blank. The proline content (µg/g FW) was quantified by the ninhydrin acid reagent method using proline as the standard [90].

### 4.5. Protein Extraction and Quantification

Freshly harvested rice leaves were ground with a mortar and pestle that was pre-chilled with liquid nitrogen. Total proteins were extracted from ground plant tissues with the trichloroacetic acid (TCA)-acetone precipitation method [91]. Protein concentrations were estimated using Bradford’s reagent [87].

### 4.6. Isoelectric Focusing (IEF) and SDS–PAGE Conditions

For each sample, 500 μg of protein was mixed with rehydration buffer (7 M urea, 2 M thiourea, 4% (*w*/*v*) 3-((3-Cholamidopropyl)dimethylammonium)-1-propanesulfonate (CHAPS), 65 mM Dithiothreitol (DTT), 0.2% Bio-Lyte, and 0.001% (*w*/*v*) bromphenol blue) to a total volume of 330 μL. Protein samples were absorbed into 17-cm pH 5–8 Bio-Rad Ready Gel Strips (Bio-Rad, Hercules, USA) following the manufacturer’s instructions. The IEF steps were 250 V for 1 h, 500 V for 1 h, 1000 V for 1 h, 1000–10,000 V for 5 h with a linear gradient, 10,000 V for 6.5 h, and a 500 V hold using a PROTEAN IEF machine (Bio-Rad). After IEF, the strips were incubated in equilibration buffer I (6 M urea, 2% SDS, 0.375 M Tris-HCl (pH 8.8), 20% glycerol, and 2% dithiothreitol) for 15 min and then in equilibration buffer II (6 M urea, 2% SDS, 0.375 M Tris-HCl (pH 8.8), 20% glycerol, and 2.5% iodoacetamide) for 15 min. After equilibration, the strips were placed on the 2nd-dimension gel and sealed with 1% agarose. For second-dimension electrophoresis, the equilibrated strips were transferred to 12% SDS-polyacrylamide gels with 5% stacking gels sealed with 1% agarose. Electrophoresis (2D) was performed at 1 W for the first 45 min and then 12 W until the bromophenol blue dye reached the bottom of the gel using a PROTEAN II XL machine (Bio-Rad, Hercules, USA).

### 4.7. Protein Visualization and Gel-Image Analysis

Protein spots were visualized by staining with Coomassie Brilliant Blue G-250. Three biological replicates for each treatment were analyzed and scanned with a UMAX PowerLook Scanner (Bio-Rad, Hercules, CA, USA). Protein spots were quantified by PDQuest 8.0 (Bio-Rad) using the “total density in gel image” method to normalize spot quantities between gels. Saturated spots were excluded from the analysis. Student’s *t*-tests (*p* < 0.05) were used to determine significant differences between treated samples and controls. Only spots that showed statistically significant differences in intensities were selected for analysis.

### 4.8. Protein In-Gel Digestion

Proteins spots with a fold change ≥ 2.0 and quality score > 80 (*p* < 0.05) were manually excised from selected gels. Then, the gel slices were washed with 500 μL of double-distilled water and then destained three times with 500 μL of 100 mmol/L ammonium bicarbonate in 50% acetonitrile for 1 h per wash on a mixer. Supernatants were discarded after each washing step. The gel spots were dehydrated by the addition of 500 μL 100% acetonitrile (ACN). Disulfide bonds were cleaved by incubating the samples for 1 h at 56 °C with 200 μL of 10 mmol/L DTT in 100 mmol/L ammonium bicarbonate buffer. The alkylation of cysteine was performed by adding 200 μL of 55 mmol/L iodoacetamide in 100 mmol/L ammonium bicarbonate buffer and by incubating for 45 min at room temperature in darkness. Gel particles were washed twice with 100 mmol/L ammonium bicarbonate buffer and dehydrated with 500 μL of acetonitrile. Gel slices were covered with trypsin solution (10 ng/μL in 100 mmol/L ammonium bicarbonate buffer). After incubation for 30 min on ice, the remaining trypsin solution was removed and 25 μL of 100 mmol/L ammonium bicarbonate was added. Proteolysis was performed at 37 °C overnight (at least >6 h). The tryptic peptides were extracted from the gel particles with 5% formic acid in 50% acetonitrile 3 times. The solution containing eluted peptide was concentrated up to drying by vacuum centrifugation, and the resultant extracts were analyzed by mass spectrometry.

### 4.9. MALDI-TOF-TOF MS Analysis and Database Query

The tryptic peptides were analyzed by matrix-assisted laser desorption/ionization time-of-flight tandem mass spectrometry (MALDI-TOF-TOF MS) with minor modifications in positive ion reflector mode. Prior to placement on the target plate, the peptides were eluted directly with 1 μL matrix solution (a-cyano-4-hydroxy-cinnamic acid in 5% TFA, 50% acetonitrile). Samples were allowed to air-dry and were analyzed by a 4700 MALDI-TOF-TOF analyzer (Applied Biosystems, Foster City, CA, USA). MS/MS spectra were acquired in the reflection mode with 0–4000 m/z by a 4700 Proteomics analyzer (Applied Bio-Systems, Framingham, MA, USA). Spectra were calibrated using trypsin autolysis products as internal standards. The acquired MS and MS/MS spectra were used to identify differentially regulated proteins using the Mascot program (http//www.martixscience.com) against *Viridiplantae* within the NCBInr protein databases (http://www.ncbi.nlm.nih.gov/). In the Mascot search, carbamidomethyl of cysteines was set as a fixed modification and oxidation of methionine was set as a variable modification. Mascot was used with the monoisotopic mass selected, a peptide mass tolerance of 100 ppm, and a fragment iron mass tolerance of 0.2 Da. Trypsin was specified as the proteolytic enzyme with one potential missed cleavage. Identification required Mascot scores higher than 73 (Mascot probability *p* < 0.05). All proteins identified by high-scoring peptides were considered true matches with at least two peptide matches. Protein hits were validated if the identification involved at least 10 top-ranking peptides with *p* < 0.05 and selected false positive rate < 0.05. When those peptides matched multiple members of a protein family, the presented protein was selected based on the highest score with at least two peptide matching.

### 4.10. DEP Identification, Annotation, and Subcellular Localization

Differentially abundant proteins were further functionally annotated using Phytozome v11.0 with Sorghum bicolor v3.1 (https://phytozome.jgi.doe.Gov/pz/portal.Html#!search?show=BLAST&method=Org_Sbicolor) genome annotation project databases and SIB Bioinformatic resource portal (http://www.expasy.org/proteomics) with UniProtKB Complete proteome (http://www.uniprot.org/) annotation project database. Subcellular location was identified using SIB Bioinformatic resource portal (http://www.expasy.org/proteomics) with UniProtKB Complete proteome (http://www.uniprot.org/) annotation project databases.

### 4.11. Statistical Analysis

Physiological parameters and spot intensity data were statistically analyzed using Duncan’s multiple range test or the Student’s *t*-test of Statistical Package for the Social Sciences (SPSS 17.0, SPSS Inc., Chicago, IL, USA). All the results are presented as mean values ± standard deviation (SD) of at least three independent experiments. *p*-value ≤ 0.05 was considered statistically significant.

## 5. Conclusions

The present study sheds light on the molecular mechanisms underlying drought tolerance of sorghum using a combined physiological and proteomic approach. Generation of MDA due to drought stress indicates lipid peroxidation and cellular injury. However, plants were able to increase proline levels and antioxidant activity to cope with drought stress; the physiological mechanism of sorghum to drought was attributed to the elimination of harmful free radicals and alleviation of oxidative stress via the synergistic action of antioxidant enzymes, such as SOD, POD, and PPO. Comparative proteomic analysis revealed 52 identities (representing 49 unique proteins) differentially expressed in sorghum roots under drought stress conditions, which were determined to be involved in various molecular processes. Key pathways of carbohydrate and energy metabolism, antioxidant and defense response, protein synthesis/processing/degradation, transcriptional regulation, amino acid biosynthesis, and nitrogen metabolism likely contribute jointly to the outstanding drought tolerance seen in sorghum. The drought tolerant C_4_ plant root proteome analysis indicated that the combined activities of several protein groups may enable the plants to tolerate drought stress. The efficient mechanisms for changes in energy usage, osmotic adjustment, ROS scavenging, protein synthesis, processing, and proteolysis play important roles for maintaining root growth as the drought stress. In addition, our elucidation of the stress-related proteins presented in roots of sorghum may help to provide new insights into drought tolerance mechanisms and valuable information in C4 plants toward improving drought tolerance of cereals.

## Figures and Tables

**Table 1:** Average number of protein spots revealed after 2-DE of sorghum seedlings treated with 10% PEG-6000 at 24 h posttreatment: the total numbers of up- or downregulated spots were obtained after matching between control (CK) and treated with 10% PEG-6000 gels. The results are means of three independent replicates.

	*Mock*	*PEG* Treated
Total number of spots	906	653
Replicates		494
Up-regulated (Quality score > 80 and fold > 2.0)		43
Up-regulated (Quality score < 80 and fold > 2.0)		17
Down-regulated (Quality score > 80 and fold > 2.0)		22
Down-regulated (Quality score < 80 and fold > 2.0)		23
Other spots (fold < 2.0)		389

**Table 2:** Upregulated protein spots in root of sorghum seedlings treated with PEG-6000 imitation drought stress at 24 h posttreatment (hpt) identified by Peptide Mass Fingerprint (PMF) query: the criteria of selection for identification was 2.0 fold change or greater change compared to the mock and a Mascot quality score of over 80. All values are means from three independent experiments.

Spot No. (ssp) ^a^	Protein Function ^b^	Subcellular Location ^c^	Mock Average Qty	PEG-6000 Treated Average Qty	Fold Change ^d^
0004	abscisic acid-activated signaling pathway, defense response	cytoplasm, nucleus	2417.1 ± 389.0	5547.8 ± 755.3	2.30
0007	failed to identify spot	unknown	384.5 ± 38.5	811.6 ± 47.9	2.11
0008	superoxide metabolic process, toxin catabolic process, oxidation-reduction process	apoplast, chloroplast stroma, cytoplasm, extracellular space, thylakoid	367.2 ± 15.4	766.1 ± 89.6	2.09
0010	failed to identify	unknown	N	Y	≥10
0114	response to oxidative stress, hydrogen peroxide catabolic process	extracellular region or secreted	408.3 ± 45.5	823.5 ± 65.1	2.02
0117	failed to identify	unknown	203.1 ± 11.2	527.9 ± 20.6	2.62
0212	intracellular protein transport, membrane fusion	snare complex, vacuolar membrane	375.6 ± 25.6	779.5 ± 46.0	2.08
0313	racemase and epimerase activity, acting on amino acids and derivatives	unknown	283.8 ± 23.7	570.5 ± 36.9	2.01
0508	failed to identify	unknown	N	Y	≥10
1104	phosphorelay signal transduction system	intracellular	145.2 ± 8.2	457.7 ± 37.0	3.15
1109	transcription, transcription regulation	nucleus	395.2 ± 7.7	831.7 ± 52.5	2.10
2001	failed to identify	unknown	837.8 ± 24.6	1780.5 ± 35.8	2.13
2004	failed to identify	unknown	345.7 ± 26.1	715.2 ± 65.3	2.07
2005	protein biosynthesis	cytoplasm	257.9 ± 17.6	1441.4 ± 95.9	5.59
2107	actin binding	unknown	165.8 ± 14.0	721.3 ± 64.6	4.35
2113	failed to identify	unknown	338.8 ± 14.3	686.9 ± 53.1	2.03
2210	unknown	unknown	182.5 ± 13.8	1803.4 ± 126.4	9.88
2602	glucose catabolic process, cellular carbohydrate metabolic process	cytoplasm	737.7 ± 23.1	1511.4 ± 153.0	2.05
2604	glucose catabolic process, cellular carbohydrate metabolic process	cytoplasm	1770.5 ± 104.4	3642.4 ± 446.6	2.06
3006	protein biosynthesis	cytoplasm	697.0 ± 12.2	1415.6 ± 65.7	2.03
3203	glyoxylate cycle	cytoplasm	130.5 ± 21.2	336.5 ± 46.0	2.58
3716	carboxylyase activity, thiamine pyrophosphate	cytosol	337.3 ± 65.4	1496.2 ± 85.5	4.44
4001	posttranscriptional gene silencing by RNA, gene silencing by RNA	nucleus, cytoplasm, risc complex,	186.8 ± 8.5	392.0 ± 18.2	2.10
4107	fail to identify	unknown	243.4 ± 19.1	494.5 ± 23.0	2.03
4403	formaldehyde catabolic process	cytosol	1132.8 ± 182.3	3760.6 ± 206.4	3.32
4511	regulation of abscisic acid biosynthetic process	unknown	143.5 ± 11.0	329.3 ± 31.9	2.29
4815	glycosyltransferase, sucrose metabolic process	plasma membrane, vacuole	149.0 ± 14.4	313.6 ± 31.7	2.10
5001	nitrite reductase [NAD(P)H] activity, nitrate assimilation	chloroplast envelope, chloroplast thylakoid membrane	312.5 ± 19.6	628.9 ± 64.1	2.01
5110	failed to identify	unknown	110.5 ± 6.2	222.2 ± 29.0	2.01
5516	lipid metabolic process, lipid degradation	cytoplasm	249.4 ± 11.6	506.6 ± 18.3	2.03
5608	oxidoreductase activity, acting on the aldehyde or oxo group of donors, NAD or NADP as acceptor	cytoplasm, membrane	82.2 ± 12.4	311.5 ± 56.9	3.79
6102	ammonia assimilation cycle, glutamate biosynthetic process	cytoplasm	111.4 ± 11.8	232.3 ± 30.6	2.09
6108	response to stress	unknown	171.1 ± 23.6	538.3 ± 61.9	3.15
6206	glycolysis, fructose 6-phosphate metabolic process	cytoplasm	158.5 ± 15.3	325.9 ± 16.7	2.06
6602	glycolysis, fructose 6-phosphate metabolic process	cytoplasm	385.3 ± 10.3	1041.5 ± 177.1	2.70
6608	UDP-N-acetylglucosamine biosynthetic process	cytoplasm	125.6 ± 16.8	253.9 ± 26.1	2.02
6815	fail to identify	unknown	42.3 ± 2.8	206.5 ± 13.6	4.90
7003	fail to identify	unknown	140.3 ± 16.9	281.4 ± 25.7	2.01
7507	ribonucleoprotein, translation	small ribosomal subunit, chloroplast	N	Y	≥10
7806	oxylipin biosynthetic process, fatty acid biosynthesis, fatty acid metabolism, lipid biosynthesis, lipid metabolism	chloroplast	240.1 ± 22.1	483.5 ± 32.3	2.01
8506	oxidation-reduction process, response to oxidative stress, hydrogen peroxide catabolic process	peroxisome, plasma membrane, cytoplasm	152.1 ± 17.1	1006.0 ± 24	6.60
8602	response to drought stress	cytoplasm	N	Y	≥10
8717	unknown	unknown	153.2 ± 14.9	1520.0 ± 133.9	9.92

a. *ssp*: Automatic allocation of protein serial number by PDQuest, spot no: spot number. b. Function were identified using Phytozome v11.0 with *Sorghum bicolor* v3.1 (https://phytozome.jgi.doe.gov/pz/portal.html#!search?show=BLAST&method=Org_Sbicolor) genome annotation project databases and SIB Bioinformatic resource portal (http://www.expasy.org/proteomics) with UniProtKB Complete proteome (http://www.uniprot.org/) annotation project database. c. Subcellular location was identified using SIB Bioinformatic resource portal (http://www.expasy.org/proteomics) with UniProtKB Complete proteome (http://www.uniprot.org/) annotation project databases. d. Fold change = PEG-treated average Qty/Mock average Qty. Qty: Normalized protein spot quantity.

**Table 3:** Downregulated protein spots in root of sorghum seedlings treated with PEG-6000 imitation drought stress at 24 h posttreatment identified by Peptide Mass Fingerprint (PMF) query: the criteria of selection for identification was a 0.5-fold or lower change compared to the mock and a Mascot quality score of over 80. All values are means from three independent experiments.

Spot No (ssp) ^a^	Protein Function ^b^	Subcellular Location ^c^	Mock Average Qty	PEG-6000 Treated Average Qty	Fold Change ^d^
0403	gluconeogenesis, glycolytic process	cytosol	298.5 ± 17.2	145.3 ± 11.6	0.49
1903	ubiquitin-dependent protein catabolic process	nucleus, cytoplasm	395.2 ± 30.2	116.8 ± 12.1	0.30
2220	cysteine biosynthetic, amino-acid biosynthesis	cytoplasm	736.5 ± 71.1	357.3 ± 36.3	0.49
3010	photosynthesis	extrinsic component of membrane, photosystem II oxygen evolving complex	Y	N	≤0.1
3110	protein serine/threonine phosphatase activity, protein dephosphorylation	cytoplasm	219.3 ± 19.5	108.6 ± 13.0	0.50
4204	amino-acid biosynthesis, cysteine biosynthesis	cytoplasm	825.4 ± 82.6	401.7 ± 25.7	0.49
5412	GDP-mannose 3,5-epimerase activity, steroid biosynthetic process,	cytoplasm	2592.4 ± 135.8	1237.1 ± 45.5	0.48
5513	pentose-phosphate shunt, gluconate utilization, oxidation-reduction process	cytosol	817.1 ± 20.0	405.4 ± 37.4	0.50
6113	glutamine metabolic process	cytosol	Y	N	≤0.1
6207	UDP-rhamnose and dTDP-rhamnose biosynthetic process	apoplast, cytosol, plasma membrane, plasmodesma	411.5 ± 32.1	202.8 ± 10.9	0.49
6311	protein biosynthesis	cytoplasm	784.0 ± 79.3	382.1 ± 23.3	0.49
6702	Hsp90 protein binding	cytoplasm	2373.8 ± 285.7	1186.0 ± 171.2	0.50
6703	oxidation-reduction process, stress response	chloroplast stroma, mitochondrion	460.0 ± 26.3	225.5 ± 13.3	0.49
6704	aspartyl-tRNA aminoacylation, protein biosynthesis	cytoplasm	799.6 ± 74.3	396.2 ± 39.5	0.50
6816	fail to identify	unknown	719.1 ± 40.1	355.6 ± 24.1	0.49
7105	fail to identify	unknown	718.0 ± 46.2	355.8 ± 23.6	0.50
7115	a lipid bilayer along with all the proteins and protein complexes embedded in it an attached to it	mitochondrion inner membrane	Y	N	≤0.1
7215	plant secondary metabolism	apoplast	Y	N	≤0.1
7407	regulation of RNA metabolic process	nucleolus	663.8 ± 40.2	327.2 ± 13.9	0.49
7409	oxidation-reduction process, cellular amino acid metabolic process	mitochondrion, vacuolar membrane	1261.4 ± 181.5	281.9 ± 26.6	0.22
7705	purine nucleobase biosynthetic process, 10-formyltetrahydrofolate biosynthetic process	cytoplasm	335.4 ± 12.2	164.3 ± 2.3	0.49
8204	siderophore biosynthetic process, oxidation-reduction process	cytosol	907.6 ± 58.2	431.6 ± 25.7	0.48

a. Automatic allocation of protein serial number by PDQuest. b. Function were identified using Phytozome v11.0 with *Sorghum bicolor* v3.1 (https://phytozome.jgi.doe.gov/pz/portal.html#!search?show=BLAST&method=Org_Sbicolor) genome annotation project databases and SIB Bioinformatic resource portal (http://www.expasy.org/proteomics) with UniProtKB Complete proteome (http://www.uniprot.org/) annotation project database. c. Subcellular location were identified using SIB Bioinformatic resource portal (http://www.expasy.org/proteomics) with UniProtKB Complete proteome (http://www.uniprot.org/) annotation project databases. d. Fold change = PEG-treated average Qty/Mock average Qty.

**Table 4:** Protein identification from 2-DE gels by peptide mass fingerprint.

Spot No (ssp) ^a^	Protein Identification ^b^	Mascot Score ^c^	Sequence Coverage (%) ^d^	Estimated Mw(kDa)/PI ^e^	Experimental Mw(kDa)/PI ^f^	Gene/Locus ^g^	Accession No ^h^	Taxonomy ^i^
0004	Bet_v_1 domain-containing protein	137	76	17.1/5.05	15.6/5.3	*Sb01g037940*	XP_002468006	*Sorghum bicolor*
0008	Superoxide dismutase (Cu-Zn SOD EC = 1.15.1.1)	81	42	20.8/5.30	16.6/5.45	*Sb07g023950*	XP_002445671	*Sorghum bicolor*
0114	Peroxidase (EC = 1.11.1.7)	114	45	36.8/4.99	30.4/5.53	*Sb06g033850*	XP_002448823	*Sorghum bicolor*
0212	Uncharacterized protein	210	68	32.6/5.04	32.4/5.54	*Sb07g018430*	XP_002444250	*Sorghum bicolor*
0313	MR_MLE domain-containing protein	80	37	46.0/5.10	40.5/5.40	*Sb03g006670*	XP_002455229	*Sorghum bicolor*
0403	Phosphoglycerate kinase (EC = 2.7.2.3)	135	45	50.2/5.89	41.5/5.41	*Sb09g024340*	XP_002441313	*Sorghum bicolor*
1104	Uncharacterized protein	105	50	19.0/5.28	25.2/5.65	*N/A*	ABK21830	*Picea sitchensis*
1109	NAC domain-containing protein	73	30	35.6/6.54	27.9/5.83	*Sb05g001590*	XP_002448920	*Sorghum bicolor*
1903	UBA_e1_C domain-containing protein	247	42	117.7/5.18	111.7/5.7	*Sb08g000540*	XP_002442655	*Sorghum bicolor*
2005	Eukaryotic translation initiation factor 5A (eIF-5A)	92	50	17.7/5.61	18.5/6.02	*TIF5A*	NP_001105606	*Zea mays*
2107	NAB domain-containing protein	84	17	105.9/5.30	29.9/6.03	*Sb03g047500*	XP_002457032	*Sorghum bicolor*
2210	Putative uncharacterized protein	90	18	122.4/6.05	33.0/6.00	*Sb07g001185*	XP_002443741	*Sorghum bicolor*
2220	Cysteine synthase	186	59	42.3/8.48	34.7/5.9	*Sb03g009260*	XP_002457554	*Sorghum bicolor*
2602	Uncharacterized protein	134	43	60.4/5.41	64.8/5.92	*Sb03g038020*	KXG33615	*Sorghum bicolor*
2604	Uncharacterized protein	147	38	60.4/5.41	64.86/6.01	*Sb03g038020*	KXG33615	*Sorghum bicolor*
3006	Eukaryotic translation initiation factor 5A(eIF-5A)	117	59	17.7/5.61	19.0/6.30	*TIF5A*	NP_001105606	*Zea mays*
3010	PsbP domain-containing protein	107	47	27.7/8.63	24.4/6.24	*Sb02g002690*	XP_002461438	*Sorghum bicolor*
3110	Probable protein phosphatase 2C 76 isoform X2	86	31	39.4/5.55	30.4/6.13	*LOC105115317*	XP_011010457	*Populus euphratica*
3203	Uncharacterized protein	98	41	33.5/5.43	34.1/6.16	*Sb06g013750*	XP_002447699	*Sorghum bicolor*
3716	Uncharacterized protein	187	52	66.4/5.67	69/6.30	*Sb01g038360*	XP_002465414	*Sorghum bicolor*
4001	Ribonuclease	104	20	100/6.82	15.4/6.38	*Sb04g021280*	XP_002453908	*Sorghum bicolor*
4204	Cysteine synthase	96	36	28.3/5.86	35.6/6.4	*Sb08g022280*	KXG24064	*Sorghum bicolor*
4403	Uncharacterized protein	149	67	41.9/5.72	43.9/6.43	*Sb05g009350*	XP_002449392	*Sorghum bicolor*
4511	Uncharacterized protein	156	53	44.9/5.72	49.5/6.52	*Sb01g043260*	XP_002465659	*Sorghum bicolor*
4815	Sucrose synthase EC = 2.4.1.13	111	18	92.1/5.82	94.2/6.50	*Sb10g006330*	ACM69042	*Sorghum bicolor*
5001	Rieske-like [2Fe-2S] domain containing protein	75	30	29.8/5.49	17.1/6.62	*RADI4G21270*	XP_003577663	*Brachypodium distachyon*
5412	Epimerase domain-containing protein	138	39	43.3/5.94	46.8/6.6	*Sb01g021890*	XP_002467242	*Sorghum bicolor*
5513	6-phosphogluconate dehydrogenase, decarboxylating (EC = 1.1.1.44)000	97	23	54.3/6.00	53.6/6.67	*Sb05g016740*	XP_002449496	*Sorghum bicolor*
5516	Patatin (EC:3.1.1.-)	78	22	47.0/6.27	49.0/6.70	*Sb07g023210*	XP_002445639	*Sorghum bicolor*
5608	Mitochondrial aldehyde dehydrogenase	97	23	59.5/6.65	57.9/6.67	*Sb10g009790*	BAB92019	*Sorghum bicolor*
6102	Glutamine amidotransferase type-2 domain-containing protein	74	9	235.8/5.49	29.8/6.73	*Sb03g031310*	XP_002458326	*Sorghum bicolor*
6108	Uncharacterized protein	126	64	29.7/8.33	25.8/6.81	*Sb01g039360*	XP_002465466	*Sorghum bicolor*
6113	DUF3700 domain-containing protein	112	47	28.2/6.95	28.8/6.77	*Sb06g033080*	XP_002448783	*Sorghum bicolor*
6206	Pyrophosphate--fructose 6-phosphate 1-phosphotransferase subunit beta	110	29	61.7/6.07	32.1/6.81	*Sb10g008850*	XP_002438147	*Sorghum bicolor*
6207	RmlD_sub_bind domain-containing protein	106	46	34.2/6.15	33.6/6.82	*Sb04g031900*	XP_002454480	*Sorghum bicolor*
6311	Eukaryotic translation initiation factor 3 subunit I(eIF3i)	131	47	36.3/6.06	40.8/6.89	*Sb02g003760*	XP_002461510	*Sorghum bicolor*
6602	Pyrophosphate--fructose 6-phosphate 1-phosphotransferase subunit beta	239	58	61.7/6.07	66.6/6.73	*Sb10g008850*	XP_002438147	*Sorghum bicolor*
6608	Uncharacterized protein	152	45	54.8/6.08	59.9/6.81	*Sb06g028340*	KXG27103	*Sorghum bicolor*
6702	Uncharacterized protein	283	64	65.4/6.07	80/6.75	*Sb04g033510*	XP_002454564	*Sorghum bicolor*
6703	Pyr_redox_2 domain-containing protein	74	26	51.6/8.57	76.7/6.77	*Sb07g003280*	KXG24413	*Sorghum bicolor*
6704	Aspartate--tRNA ligase 2 cytoplasmic	163	31	61.2/5.92	70.9/6.79	*LOC100279253*	NP_001145746	*Zea mays*
7115	Prohibitin	101	44	30.7/6.55	30.8/7.06	*Sb06g019110*	XP_002447973	*Sorghum bicolor*
7215	Dirigent protein	126	47	32.6/6.75	31.6/7.06	*Sb09g001880*	XP_002439182	*Sorghum bicolor*
7407	Heterogeneous nuclear ribonucleoprotein 1	86	30	44.2/6.17	47.0/7.01	*LOC100382683*	NP_001168878	*Zea mays*
7409	Glutamate dehydrogenase	146	48	44.8/6.24	72.6/7.03	*Sb06g024150*	XP_002446878	*Sorghum bicolor*
7507	Small ribosomal protein 4 (rps4)	77	47	23.0/10.9	48.5/7.26	*rps4*	AAL26212	*Pinus thunbergii*
7705	Uncharacterized protein	119	28	81.0/8.50	74.1/7.15	*Sb02g026140*	OQU89595	*Sorghum bicolor*
7806	Lipoxygenase (EC = 1.13.11.58)	109	24	100.6/6.26	104.6/6.85	*Sb01g011040*	XP_002466613	*Sorghum bicolor*
8204	Aldo_ket_red domain-containing protein	203	61	39.6/8.44	33.8/7.4	*Sb01g041640*	XP_002468202	*Sorghum bicolor*
8506	Catalase EC = 1.11.1.6	193	54	57.3/6.62	52.5/7.43	*Sb10g030840*	XP_002437631	*Sorghum bicolor*
8602	LEA-like protein	94	42	24.2/4.82	66.9/7.4	*LP28*	BAB97392	*Lilium longiflorum*
8717	Uncharacterized protein	153	33	81.5/6.82	79.0/7.68	*Sb06g000930*	XP_002446045	*Sorghum bicolor*

a. Automatic allocation of protein serial number by PDQuest. b,h,i. Estimates based on NCBInr by Mascot procedure (in the Viridiplantae library). c. Scores greater than 73 were considered significant (*p* < 0.05). d. The highest matching value of sequence coverage. e. Calculated by MS. f. Estimates based on 2D gel data. g. Gene locus were identified using Phytozome *v*11.0 with *Sorghum bicolor v3.1* (https://phytozome.jgi.doe.gov/pz/portal.html#!search?show=BLAST&method=Org_Sbicolor) genome annotation project databases and SIB Bioinformatic resource portal (http://www.expasy.org/proteomics) with UniProtKB Complete proteome (http://www.uniprot.org/) annotation project database. Mw: Molecular weight; PI; Protein isoelectric point.

**Table 5:** The 52 identified proteins in the root of sorghum seedlings within functional categories based on their biological functions and subcellular localization.

Categories	Classification Basis	Protein Spot Serial Number (Total 52)
Biological function	Energy and Carbohydrate metabolism	2602, 2604, 3203, 3716, 4815, 6206, 6602, 6608 0403, 3010, 5513, 6207
	Protein synthesis/processing/degradation	1903, 3110, 6311, 6704, 0212, 2005, 3006, 7507
	Antioxidant and defense response	0008, 0114, 6108, 8506, 8602, 4403, 5608, 6702, 6703, 8204
	Amino acid biosynthesis	0313, 2220, 4204, 6113, 7409 7705
	Transcription and regulation	1109, 4001, 7407
	Nitrogen metabolism	5001, 6102
	Signal transduction	0004, 1104
	Lipid membrane metabolism	5412, 7115, 5516, 7806
	Other functional categories	2107, 4511, 8717, 7215, 2210
Subcellular localization	Cytoplasm	0004, 0008, 1903, 2005, 2220, 2602, 2604, 3006, 3110, 3203, 4001, 4204, 5412, 5516, 5608, 6102, 6206, 6602, 6608, 6704, 7705, 8602
	Cytosol	0403, 3716, 4403, 5513, 6113, 6207, 8204
	Chloroplast	0008, 5001, 7507, 7806
	Chloroplast thylakoid membrane	0008, 5001
	Nucleus	0004, 1109, 1903, 4001, 7407
	Vacuolar membrane	0212, 4815, 7409
	Plasma membrane	3010, 4815, 5608, 8506, 6207
	Ribosome	7507
	Extracellular	0008, 0114, 2210, 4511, 6108
	Intracellular	1104
	Peroxisome	8506
	Chloroplast stroma	6703
	Mitochondrion	6703, 7115, 7409
	Apoplast	6207, 7215
	Uncharacterized	0313, 2107, 8717

## References

[1] Bian Y.W., Deng X., Yan X., Zhou J.X., Yuan L.L., Yan Y.M. (2017). Integrated proteomic analysis of Brachypodium distachyon roots and leaves reveals a synergistic network in the response to drought stress and recovery. Sci. Rep..

[2] Boyer J.S. (1982). Plant productivity and environment. Science.

[3] Pennisi E. (2008). Plant genetics: The blue revolution, drop by drop, gene by gene. Science.

[4] Shao H.B., Chu L.Y., Jaleel C.A., Manivannan P., Panneerselvam R., Shao M.A. (2009). Understanding water deficit stress-induced changes in the basic metabolism of higher plants—Biotechnologically and sustainably improving agriculture and the ecoenvironment in arid regions of the globe. Crit. Rev. Biotechnol..

[5] Breshears D.D., Cobb N.S., Rich P.M., Price K.P., Allen C.D., Balice R.G., Romme W.H., Kastens J.H., Floyd M.L., Belnap J. (2005). Regional vegetation die-off in response to global-change-type drought. Proc. Natl. Acad. Sci. USA.

[6] Bartels D., Furini A., Ingram J., Salamini F. (1996). Responses of plants to dehydration stress: A molecular analysis. Plant Growth Regul..

[7] Gowda V.R.P., Henry A., Yamauchi A., Shashidhar H.E., Serraj R. (2011). Root biology and genetic improvement for drought avoidance in rice. Field Crop. Res..

[8] Atkinson N.J., Urwin P.E. (2012). The interaction of plant biotic and abiotic stresses: From genes to the field. J. Exp. Bot..

[9] Fujita M., Fujita Y., Noutoshi Y., Takahashi F., Narusaka Y., Yamaguchi-Shinozaki K., Shinozaki K. (2006). Crosstalk between abiotic and biotic stress responses: A current view from the points of convergence in the stress signaling networks. Curr. Opin. Plant Biol..

[10] Dietz K.J., Mittler R., Noctor G. (2016). Recent Progress in Understanding the Role of Reactive Oxygen Species in Plant Cell Signaling. Plant Physiol..

[11] Petricka J.J., Schauer M.A., Megraw M., Breakfield N.W., Thompson J.W., Georgiev S., Soderblom E.J., Ohler U., Moseley M.A., Grossniklaus U. (2012). The protein expression landscape of the Arabidopsis root. Proc. Natl. Acad. Sci. USA.

[12] Zeng W.J., Peng Y.L., Zhao X.Q., Wu B.Y., Chen F.Q., Ren B., Zhuang Z.L., Gao Q.H., Ding Y.F. (2019). Comparative Proteomics Analysis of the Seedling Root Response of Drought-sensitive and Drought-tolerant Maize Varieties to Drought Stress. Int. J. Mol. Sci..

[13] Labuschagne M.T. (2018). A review of cereal grain proteomics and its potential for sorghum improvement. J. Cereal Sci..

[14] Duodu K.G., Taylor J.R.N., Belton P.S., Hamaker B.R. (2003). Factors affecting sorghum protein digestibility. J. Cereal Sci..

[15] Borrell A.K., Mullet J.E., George-Jaeggli B., van Oosterom E.J., Hammer G.L., Klein P.E., Jordan D.R. (2014). Drought adaptation of stay-green sorghum is associated with canopy development, leaf anatomy, root growth, and water uptake. J. Exp. Bot..

[16] Ngara R., Ndimba B.K. (2014). Understanding the complex nature of salinity and drought-stress response in cereals using proteomics technologies. Proteomics.

[17] Kailasa S.K., Wu H.F. (2014). Recent developments in nanoparticle-based MALDI mass spectrometric analysis of phosphoproteomes. Microchim. Acta.

[18] Ghosh D., Xu J. (2014). Abiotic stress responses in plant roots: A proteomics perspective. Front. Plant Sci..

[19] Vincent D., Lapierre C., Pollet B., Cornic G., Negroni L., Zivy M. (2005). Water deficits affect caffeate O-methyltransferase, lignification, and related enzymes in maize leaves. A proteomic investigation. Plant Physiol..

[20] Jorge I., Navarro R.M., Lenz C., Ariza D., Jorrin J. (2006). Variation in the holm oak leaf proteome at different plant developmental stages, between provenances and in response to drought stress. Proteomics.

[21] Alam I., Sharmin S.A., Kim K.H., Yang J.K., Choi M.S., Lee B.H. (2010). Proteome analysis of soybean roots subjected to short-term drought stress. Plant Soil.

[22] Sengupta D., Kannan M., Reddy A.R. (2011). A root proteomics-based insight reveals dynamic regulation of root proteins under progressive drought stress and recovery in *Vigna radiata* (L.) Wilczek. Planta.

[23] Salekdeh G.H., Siopongco J., Wade L.J., Ghareyazie B., Bennett J. (2002). Proteomic analysis of rice leaves during drought stress and recovery. Proteomics.

[24] Hu Y., Li W.C., Xu Y.Q., Li G.J., Liao Y., Fu F.L. (2009). Differential expression of candidate genes for lignin biosynthesis under drought stress in maize leaves. J. Appl. Genet..

[25] Muthurajan R., Shobbar Z.S., Jagadish S.V.K., Bruskiewich R., Ismail A., Leung H., Bennett J. (2011). Physiological and Proteomic Responses of Rice Peduncles to Drought Stress. Mol. Biotechnol..

[26] Ashoub A., Beckhaus T., Berberich T., Karas M., Bruggemann W. (2013). Comparative analysis of barley leaf proteome as affected by drought stress. Planta.

[27] Yang L.M., Jiang T.B., Fountain J.C., Scully B.T., Lee R.D., Kemerait R.C., Chen S.X., Guo B.Z. (2014). Protein Profiles Reveal Diverse Responsive Signaling Pathways in Kernels of Two Maize Inbred Lines with Contrasting Drought Sensitivity. Int. J. Mol. Sci..

[28] Qin N., Xu W.G., Hu L., Li Y., Wang H.W., Qi X.L., Fang Y.H., Hua X. (2016). Drought tolerance and proteomics studies of transgenic wheat containing the maize C4 phosphoenolpyruvate carboxylase (PEPC) gene. Protoplasma.

[29] Li Z., Li Z., Muhammad W., Lin M.H., Azeem S., Zhao H., Lin S., Chen T., Fang C.X., Letuma P. (2018). Proteomic analysis of positive influence of alternate wetting and moderate soil drying on the process of rice grain filling. Plant Growth Regul..

[30] Thangella P.A.V., Pasumarti S.N.B.S., Pullakhandam R., Geereddy B.R., Daggu M.R. (2018). Differential expression of leaf proteins in four cultivars of peanut (*Arachis hypogaea* L.) under water stress. 3 Biotech.

[31] Paterson A.H., Bowers J.E., Bruggmann R., Dubchak I., Grimwood J., Gundlach H., Haberer G., Hellsten U., Mitros T., Poliakov A. (2009). The Sorghum bicolor genome and the diversification of grasses. Nature.

[32] Abdel-Ghany S.E., Ullah F., Ben-Hur A., Reddy A.S.N. (2020). Transcriptome Analysis of Drought-Resistant and Drought-Sensitive Sorghum (*Sorghum bicolor*) Genotypes in Response to PEG-Induced Drought Stress. Int. J. Mol. Sci..

[33] Ngara R., Ndimba B.K. (2011). Mapping and characterisation of the sorghum cell suspension culture secretome. Afr. J. Biotechnol..

[34] Ngara R., Ramulifho E., Movahedi M., Shargie N.G., Brown A.P., Chivasa S. (2018). Identifying differentially expressed proteins in sorghum cell cultures exposed to osmotic stress. Sci. Rep..

[35] Fadoul H.E., El Siddig M.A., Abdalla A.W.H., El Hussein A.A. (2018). Physiological and proteomic analysis of two contrasting Sorghum bicolor genotypes in response to drought stress. Aust. J. Crop Sci..

[36] Goche T., Shargie N.G., Cummins I., Brown A.P., Chivasa S., Ngara R. (2020). Comparative physiological and root proteome analyses of two sorghum varieties responding to water limitation. Sci. Rep..

[37] Swami A.K., Alam S.I., Sengupta N., Sarin R. (2011). Differential proteomic analysis of salt stress response in Sorghum bicolor leaves. Environ. Exp. Bot..

[38] Ngara R., Ndimba R., Borch-Jensen J., Jensen O.N., Ndimba B. (2012). Identification and profiling of salinity stress-responsive proteins in Sorghum bicolor seedlings. J. Proteom..

[39] Jedmowski C., Ashoub A., Beckhaus T., Berberich T., Karas M., Bruggemann W. (2014). Comparative Analysis of Sorghum bicolor Proteome in Response to Drought Stress and following Recovery. Int. J. Proteom..

[40] Roy S.K., Cho S.W., Kwon S.J., Kamal A.M., Kim S.W., Oh M.W., Lee M.S., Chung K.Y., Xin Z.G., Woo S.H. (2016). Morpho-Physiological and Proteome Level Responses to Cadmium Stress in Sorghum. PLoS ONE.

[41] Roy S.K., Kwon S.J., Cho S.W., Kamal A.H.M., Kim S.W., Sarker K., Oh M.W., Lee M.S., Chung K.Y., Xin Z.G. (2016). Leaf proteome characterization in the context of physiological and morphological changes in response to copper stress in sorghum. Biometals.

[42] Koca H., Bor M., Ozdemir F., Turkan I. (2007). The effect of salt stress on lipid peroxidation, antioxidative enzymes and proline content of sesame cultivars. Environ. Exp. Bot..

[43] Veeranagamallaiah G., Chandraobulreddy P., Jyothsnakumari G., Sudhakar C. (2007). Glutamine synthetase expression and pyrroline-5-carboxylate reductase activity influence proline accumulation in two cultivars of foxtail millet (*Setaria italica* L.) with differential salt sensitivity. Environ. Exp. Bot..

[44] Maggio A., Miyazaki S., Veronese P., Fujita T., Ibeas J.I., Damsz B., Narasimhan M.L., Hasegawa P.M., Joly R.J., Bressan R.A. (2002). Does proline accumulation play an active role in stress-induced growth reduction?. Plant J..

[45] Claussen W. (2005). Proline as a measure of stress in tomato plants. Plant Sci..

[46] Kishor P.B.K., Sangam S., Amrutha R.N., Laxmi P.S., Naidu K.R., Rao K.R.S.S., Rao S., Reddy K.J., Theriappan P., Sreenivasulu N. (2005). Regulation of proline biosynthesis, degradation, uptake and transport in higher plants: Its implications in plant growth and abiotic stress tolerance. Curr. Sci. India.

[47] Ben Rejeb K., Abdelly C., Savoure A. (2014). How reactive oxygen species and proline face stress together. Plant Physiol. Biochem..

[48] Deeba F., Pandey A.K., Ranjan S., Mishra A., Singh R., Sharma Y.K., Shirke P.A., Pandey V. (2012). Physiological and proteomic responses of cotton (*Gossypium herbaceum* L.) to drought stress. Plant Physiol. Biochem..

[49] Horvath E., Szalai G., Janda T. (2007). Induction of abiotic stress tolerance by salicylic acid signaling. J. Plant Growth Regul..

[50] Moller I.M., Jensen P.E., Hansson A. (2007). Oxidative modifications to cellular components in plants. Annu. Rev. Plant Biol..

[51] Sharma P., Dubey R.S. (2005). Drought induces oxidative stress and enhances the activities of antioxidant enzymes in growing rice seedlings. Plant Growth Regul..

[52] Parida A.K., Das A.B. (2005). Salt tolerance and salinity effects on plants: A review. Ecotoxicol. Environ. Saf..

[53] Zorb C., Schmitt S., Muhling K.H. (2010). Proteomic changes in maize roots after short-term adjustment to saline growth conditions. Proteomics.

[54] Mirzaei M., Soltani N., Sarhadi E., George I.S., Neison K.A., Pascovici D., Shahbazian S., Haynes P.A., Atwell B.J., Salekdeh G.H. (2014). Manipulating Root Water Supply Elicits Major Shifts in the Shoot Proteome. J. Proteome Res..

[55] Huang S.B., Greenway H., Colmer T.D., Millar A.H. (2005). Protein synthesis by rice coleoptiles during prolonged anoxia: Implications for glycolysis, growth and energy utilization. Ann. Bot.-Lond..

[56] Chitteti B.R., Peng Z.H. (2007). Proteome and phosphoproteome differential expression under salinity stress in rice (*Oryza sativa*) roots. J. Proteome Res..

[57] Wang M.C., Peng Z.Y., Li C.L., Li F., Liu C., Xia G.M. (2008). Proteomic analysis on a high salt tolerance introgression strain of *Triticum aestivum*/*Thinopyrum ponticum*. Proteomics.

[58] Manaa A., Ben Ahmed H., Valot B., Bouchet J.P., Aschi-Smiti S., Causse M., Faurobert M. (2011). Salt and genotype impact on plant physiology and root proteome variations in tomato. J. Exp. Bot..

[59] Xu C.P., Sibicky T., Huang B.R. (2010). Protein profile analysis of salt-responsive proteins in leaves and roots in two cultivars of creeping bentgrass differing in salinity tolerance. Plant Cell Rep..

[60] Jiang Y., Yang B., Harris N.S., Deyholos M.K. (2007). Comparative proteomic analysis of NaCl stress-responsive proteins in Arabidopsis roots. J. Exp. Bot..

[61] Witzel K., Weidner A., Surabhi G.K., Borner A., Mock H.P. (2009). Salt stress-induced alterations in the root proteome of barley genotypes with contrasting response towards salinity. J. Exp. Bot..

[62] Peng Z.Y., Wang M.C., Li F., Lv H.J., Li C.L., Xia G.M. (2009). A Proteomic Study of the Response to Salinity and Drought Stress in an Introgression Strain of Bread Wheat. Mol. Cell Proteom..

[63] Zhou S.P., Sauve R.J., Liu Z., Reddy S., Bhatti S., Hucko S.D., Fish T., Thannhauser T.W. (2011). Identification of Salt-induced Changes in Leaf and Root Proteomes of the Wild Tomato, *Solanum chilense*. J. Am. Soc. Hortic. Sci..

[64] Zhao Q., Zhang H., Wang T., Chen S.X., Dai S.J. (2013). Proteomics-based investigation of salt-responsive mechanisms in plant roots. J. Proteom..

[65] Koh J., Chen G., Yoo M.J., Zhu N., Dufresne D., Erickson J.E., Shao H.B., Chen S.X. (2015). Comparative Proteomic Analysis of Brassica napus in Response to Drought Stress. J. Proteome Res..

[66] Xu J., Xing X.J., Tian Y.S., Peng R.H., Xue Y., Zhao W., Yao Q.H. (2015). Transgenic Arabidopsis Plants Expressing Tomato Glutathione S-Transferase Showed Enhanced Resistance to Salt and Drought Stress. PLoS ONE.

[67] Rodrigues S.M., Andrade M.O., Gomes A.P.S., DaMatta F.M., Baracat-Pereira M.C., Fontes E.P.B. (2006). Arabidopsis and tobacco plants ectopically expressing the soybean antiquitin-like ALDH7 gene display enhanced tolerance to drought, salinity, and oxidative stress. J. Exp. Bot..

[68] Sunkar R., Bartels D., Kirch H.H. (2003). Overexpression of a stress-inducible aldehyde dehydrogenase gene from Arabidopsis thaliana in transgenic plants improves stress tolerance. Plant J..

[69] Zhang H., Han B., Wang T., Chen S.X., Li H.Y., Zhang Y.H., Dai S.J. (2012). Mechanisms of Plant Salt Response: Insights from Proteomics. J. Proteome Res..

[70] Singh R., Jwa N.S. (2013). Understanding the Responses of Rice to Environmental Stress Using Proteomics. J. Proteome Res..

[71] Kosova K., Vitamvas P., Urban M.O., Prasil I.T., Renaut J. (2018). Plant Abiotic Stress Proteomics: The Major Factors Determining Alterations in Cellular Proteome. Front. Plant Sci..

[72] Hand S.C., Menze M.A., Toner M., Boswell L., Moore D. (2011). LEA Proteins During Water Stress: Not Just for Plants Anymore. Annu. Rev. Physiol..

[73] Buchanan C.D., Lim S., Salzman R.A., Kagiampakis I., Morishige D.T., Weers B.D., Klein R.R., Pratt L.H., Cordonnier-Pratt M.M., Klein P.E. (2005). Sorghum bicolor’s transcriptome response to dehydration, high salinity and ABA. Plant Mol. Biol..

[74] Dugas D.V., Monaco M.K., Olsen A., Klein R.R., Kumari S., Ware D., Klein P.E. (2011). Functional annotation of the transcriptome of Sorghum bicolor in response to osmotic stress and abscisic acid. BMC Genom..

[75] Johnson S.M., Lim F.L., Finkler A., Fromm H., Slabas A.R., Knight M.R. (2014). Transcriptomic analysis of Sorghum bicolor responding to combined heat and drought stress. BMC Genom..

[76] Zhang W., Zhang H.L., Ning L.Y., Li B., Bao M.Z. (2016). Quantitative Proteomic Analysis Provides Novel Insights into Cold Stress Responses in Petunia Seedlings. Front. Plant Sci..

[77] Zhou G., Yang L.T., Li Y.R., Zou C.L., Huang L.P., Qiu L.H., Huang X., Srivastava M.K. (2012). Proteomic Analysis of Osmotic Stress-Responsive Proteins in Sugarcane Leaves. Plant Mol. Biol. Rep..

[78] Liu C.W., Hsu Y.K., Cheng Y.H., Yen H.C., Wu Y.P., Wang C.S., Lai C.C. (2012). Proteomic analysis of salt-responsive ubiquitin-related proteins in rice roots. Rapid Commun. Mass Spectrom..

[79] Yang L., Ma C.Q., Wang L.L., Chen S.X., Li H.Y. (2012). Salt stress induced proteome and transcriptome changes in sugar beet monosomic addition line M14. J. Plant Physiol..

[80] Pang Q.Y., Chen S.X., Dai S.J., Chen Y.Z., Wang Y., Yan X.F. (2010). Comparative Proteomics of Salt Tolerance in Arabidopsis thaliana and *Thellungiella halophila*. J. Proteome Res..

[81] Britto D.T., Kronzucker H.J. (2002). NH4+ toxicity in higher plants: A critical review. J. Plant Physiol..

[82] Jacoby R.P., Millar A.H., Taylor N.L. (2010). Wheat Mitochondrial Proteomes Provide New Links between Antioxidant Defense and Plant Salinity Tolerance. J. Proteome Res..

[83] Yu J.J., Chen S.X., Zhao Q., Wang T., Yang C.P., Diaz C., Sun G.R., Dai S.J. (2011). Physiological and Proteomic Analysis of Salinity Tolerance in *Puccinellia tenuiflora*. J. Proteome Res..

[84] Ahn C.S., Lee J.H., Hwang A.R., Kim W.T., Pai H.S. (2006). Prohibitin is involved in mitochondrial biogenesis in plants. Plant J..

[85] Long R.C., Li M.N., Zhang T.J., Kang J.M., Sun Y., Cong L.L., Gao Y.L., Liu F.Q., Yang Q.C. (2016). Comparative Proteomic Analysis Reveals Differential Root Proteins in *Medicago sativa* and *Medicago truncatula* in Response to Salt Stress. Model Legume Med. Truncatula.

[86] Horie T., Motoda J., Kubo M., Yang H., Yoda K., Horie R., Chan W.Y., Leung H.Y., Hattori K., Konomi M. (2005). Enhanced salt tolerance mediated by AtHKT1 transporter-induced Na+ unloading from xylem vessels to xylem parenchyma cells. Plant. J..

[87] Bradford M.M. (1976). A rapid and sensitive method for the quantitation of microgram quantities of protein utilizing the principle of protein-dye binding. Anal. Biochem..

[88] Beyer W.F., Fridovich I. (1987). Assaying for superoxide dismutase activity: Some large consequences of minor changes in conditions. Anal. Biochem..

[89] Kwak S.S., Kim S.K., Lee M.S., Jung K.H., Park I.H., Liu J.R. (1995). Acidic Peroxidases from Suspension-Cultures of Sweet-Potato. Phytochemistry.

[90] Bates L., Waldren R., Teare I. (1973). Rapid determination of free proline for water stress studies. Plant Soil.

[91] Wang W., Scali M., Vignani R., Spadafora A., Sensi E., Mazzuca S., Cresti M. (2003). Protein extraction for two-dimensional electrophoresis from olive leaf, a plant tissue containing high levels of interfering compounds. Electrophoresis.

